# Annotations

**Published:** 1897-09-18

**Authors:** 


### Annotations.

### Insanity in Prisons.

The relation existing between insanity and crime is a matter of great interest, on the one hand as pointing to "degeneracy" as a main factor in the making of the criminal; and, on the other, in consequence of the persistently repeated assertion that our prison system conduces to insanity. The annual report of the Commissioners and Directors of Prisons for the past year shows that of the total number of insane, viz., 380, dealt with in local prisons during the year, 216 were either remanded for observation, being of doubtful sanity on committal, or they were not convicted, or they were found insane when brought to trial. So far, then, as concerns the influence of prison life in the production of insanity these cases may be left out of account. Of the remaining 164 cases, 34 were recorded as having been previously insane, and 121 were insane on reception. Only 43 out of the whole number could be fairly looked upon as cases bearing upon the question of the influence of prison life in the production of insanity. An analysis of these cases shows that, in 19 out of the 43, symptoms occurred within a month of their reception, and that out of the whole local prison population only five cases of insanity arose after the completion of six months' imprisonment. It is unnecessary to point out how strongly these figures speak in favour of the view that the treatment which the prisoners undergo is not the cause of the insanity by which as a class they are afflicted. Drink, insanity, and crime are inextricably mixed together, each aggravating the other. Hereditary taint not only causes actual insanity, but produces an even larger amount of mental instability of varying degree. In many cases it is mere chance circumstance which decides whether a man's want of control over his impulses shall first show itself by drunkenness, by thieving, by assault and battery, or by insane behaviour. Drinking and law breaking are but too often merely first symptoms of mental defect, and although that fact by no means lessens the necessity of punishing crime it certainly is sufficient to take away all surprise at the prevalence of insanity in our gaols.

### Are Athletes Healthy?

The athlete is so popular a person that we need not be surprised at the interest taken in the question, Is he healthy? If by an "athlete" is meant a "trained" man, one answer to the question is immediately obvious, viz., that whether he is healthy or not his health is not due to his training. If a demand arose, as in fact it does arise in military service, for men of endurance, of considerable muscular strength, of great and varied digestive capacity, able to bear exposure, starvation, and attacks of infectious illness with the least possible disturbance, and all the time fit for the exercise of the highest intelligence, then the training which produced such men would without doubt be conducive to health. But the training which athletes undergo does not even aim at such a condition. Its whole object is to produce the greatest possible output of muscular energy, to apply it with the utmost exactitude in a given direction, and at the same time to develop a state of body as to weight, &c., which shall conform to certain rules. The routine by which these results can be most readily obtained is, no doubt, effectual for its purpose—but that purpose is not the production of health. An athlete, then, although often a very healthy man, by no means obtains his health by virtue of his training. Quite the opposite, in fact. Those who by build and constitution require severe regimen and training to make them fit for athletic work, and who drop out of condition as soon as they cease to train, may really suffer in health from the process—in the case of jockeys and others who train for weight they often do suffer very severely—while others who are always fairly fit may even benefit by the course. But when we come to consider what, in these record breaking days, an athlete has to do both in his "sports" and in his practice (if we may draw a distinction between the practice and the regimen of his training), we are quite clear that the life of an athlete does not tend to health. We quite admit that a large number of healthy middle-aged and even old men are to be met with who have ardently indulged in athletics in their earlier years, but the fact that athletic sports are chiefly attractive to the strong and healthy makes the athletes so select a class that no comparison can be fairly drawn between them and the average man. Athletes are healthy because they are select, not because they are athletic. On the other hand, when we come to compare them with the average men we must remember that average men have many vices. We would not urge a young man to become an athlete in the modern sporting sense, but perhaps he had better even do that than oscillate between the office, the billiard-room, and the whiskey bar.

### Liberty and Sanitation.

The address delivered by Dr. Farquharson, M.P., at the opening of the Sanitary Congress at Leeds on Tuesday, was interesting in many respects, and his remarks on the parliamentary difficulties in the way of sanitary legislation may even be spoken of as important. It is certainly disheartening to find that even in such matters as the health of their constituents members of Parliament cannot put aside prejudice and work for the common good. Both sides, he declared, were bad; but that to which he belonged was the worst, for a deep-rooted suspicion of scientific methods and of progressive sanitation existed in certain Radical quarters, and abstract views of personal liberty, together with distrust of the so-called tyranny of doctors, swayed a kind of plausible sentiment which was usually irresistible in its paralysing effects upon hygienic legislation. Private members, he said, could do little but clear the way for Government action, and Government could not do much until a Ministry of Health was established to co-ordinate into one harmonious whole the scattered and perplexing threads of sanitary legislation. It is a curious and interesting thing that, while in the great democratic United States the people are willing to submit to the most autocratic dealings of Boards of Health furnished with almost absolute authority, we in England, with our customary submission to the fetish of personal liberty and private rights, hesitate and draw back from schemes which are obviously for the public good, startled by the fear of some imaginary tyranny. Ignorance objects to the rule of knowledge even in such an abstruse matter as sanitation.

